# Temperate Snake Community in South America: Is Diet Determined by Phylogeny or Ecology?

**DOI:** 10.1371/journal.pone.0123237

**Published:** 2015-05-06

**Authors:** Gisela P. Bellini, Alejandro R. Giraudo, Vanesa Arzamendia, Eduardo G. Etchepare

**Affiliations:** 1 Instituto Nacional de Limnología (CONICET-UNL), Santa Fe, Argentina; 2 Facultad de Humanidades y Ciencias, Universidad Nacional del Litoral, Santa Fe, Argentina; 3 Facultad de Ciencias Exactas y Naturales y Agrimensura, Universidad Nacional del Nordeste, Corrientes, Argentina; University of Tennessee, UNITED STATES

## Abstract

Communities are complex and dynamic systems that change with time. The first attempts to explain how they were structured involve contemporary phenomena like ecological interactions between species (e.g., competition and predation) and led to the competition-predation hypothesis. Recently, the deep history hypothesis has emerged, which suggests that profound differences in the evolutionary history of organisms resulted in a number of ecological features that remain largely on species that are part of existing communities. Nevertheless, both phylogenetic structure and ecological interactions can act together to determine the structure of a community. Because diet is one of the main niche axes, in this study we evaluated, for the first time, the impact of ecological and phylogenetic factors on the diet of Neotropical snakes from the subtropical-temperate region of South America. Additionally, we studied their relationship with morphological and environmental aspects to understand the natural history and ecology of this community. A canonical phylogenetical ordination analysis showed that phylogeny explained most of the variation in diet, whereas ecological characters explained very little of this variation. Furthermore, some snakes that shared the habitat showed some degree of diet convergence, in accordance with the competition-predation hypothesis, although phylogeny remained the major determinant in structuring this community. The clade with the greatest variability was the subfamily Dipsadinae, whose members had a very different type of diet, based on soft-bodied invertebrates. Our results are consistent with the deep history hypothesis, and we suggest that the community under study has a deep phylogenetic effect that explains most of the variation in the diet.

## Introduction

A central question to understanding how community assemblages are structured is: what determines the organization of a community in time and space? [1]. For most of the 20th century, the studies were centered on competition and predation, supporting the hypothesis that ecological interaction between species affects assemblage structure [2, 3].

Late in the 20th century, the idea that the structure of existing communities can have a significant phylogenetic influence began to develop, reflecting species interactions in the past [1, 4]. Some of the pioneering studies that addressed phylogenetic and biogeographic influences in structuring communities focused on tropical snakes of South American communities, and were published by Cadle [5] and Cadle and Greene [1]. These authors provided an alternative explanation of the structure of snake communities, previously studied in Northeast Brazil by Vitt and Vangilder [6]. Thus, the study of the ecology of Neotropical snakes has played an important role in generating two hypotheses that attempt to explain ecological differences among species currently making up communities [1, 7]. The first, called the competition—predation hypothesis (CPH), centers on recent effects, in which closely related taxa diverge to partition resources through interspecific competition or predation [8]. This hypothesis predicts that the ecological traits of coexisting species are independent of phylogeny and that major shifts in niche preference (food, time, and microhabitat) result from interactions among species within present-day assemblages [6, 7]. The CPH has been proposed as an explanation for the local community structure of Amazonian and Northeastern Brazilian snakes [6, 9].

The second hypothesis, called the deep history hypothesis (DHH), suggests that deep divergences in the evolutionary history of organisms (rather than recent effects) resulted in sets of ecological traits that are maintained for the majority of species belonging to present day assemblages [1, 7]. The DHH posits that ecological traits of coexisting species can be predicted based on phylogeny regardless of the community in which individual species currently reside [10]. Vitt and Pianka [10] investigated this possibility in a study of the diets of 12 lizard families from four continents. Colston et al. [7] revealed that snake diets are associated with seven major divergences in snake evolutionary history, based on a study of 196 species of all lineages of snakes in the world. These authors suggested that future studies should focus on the effects of these patterns on local communities around the world. Phylogenetic structure and ecological interactions may act together to determine community structure, although it is essential to identify the relative contributions of these two major forces in different organisms, regions, and species traits [7, 11].

Snakes are unique among vertebrates because of some peculiar adaptations: they are exclusively carnivorous and solitary, and obligatorily ingest their prey whole [12, 13]. Thus, they are gape-limited, and have evolved a suite of morphological and behavioural adaptations to kill their prey and ingest it whole, including sophisticated venom and the apparatus to inject it, extreme body strength and huge size to suffocate very large animals, and enormous skull distensibility [12]. In snakes, the most important sense organs involved in prey detection are eye, nose, thermal and Jacobson's organ [14, 15]. Experimental studies have shown that snakes generally exhibit precise, genetically-determined, species-specific preferences for some prey types [12, 16]. Snakes have well-developed chemosensory systems, and most species probably use chemical cues during some stage of prey recognition [17]. De Queiroz and Rodriguez-Robles [18] linked the feeding behavior of snakes, and the importance of its chemosensory system, with the evolution of diet specialization. They argued that habitat use and specific feeding traits likely are important influences on the origins of eating. Both of these characteristics can be viewed as predispositions that are the results of a lineage’s specific evolutionary history. For example, arboreality and an attraction to chemical cues produced by birds are not simply properties of any snake that finds itself in a forest with many birds but are outcomes of a contingent evolutionary pathway. Thus, the effects of such predispositions are part of the general imprint of history [1]. Other studies also have emphasized the influence of history on the ecological characteristics of communities and larger biotas [1, 4, 5, 7, 10]. Cadle and Greene [1] pointed out that differences among taxa in the tendency to exhibit a particular feeding habit can have important consequences for community composition. For example, they suggested that the lack of arthropod-eating snakes in many Neotropical communities results from the absence of snake clades with tendencies to feed on arthropods rather than from the paucity of suitable arthropods.

Despite the potential of this growing field, its importance in linking community ecology and evolutionary biology has yet to be fully realized [19]. A phylogenetic approach to studying community organization provides a new perspective of the role of competition and the maintenance of diversity in communities by highlighting the similarities of co-occurring species as well as the differences [4]. Most studies on assemblages of snakes in South America were conducted in different biomes of the tropics, and were focused primarily on the natural history of the species, only discussing some factors that structure the community locally [20]. Only two contributions, both conducted in tropical areas, have simultaneously considered ecological and phylogenetic aspects of an assemblage of South American snakes. Cadle and Greene [1], who compared fifteen rainforest assemblages, suggested that in general, all three clades of Neotropical colubrid snakes (i.e., Colubrines, Central and South American Xenodontines) include many species that consume frogs and lizards, whereas in the clade of South American Xenodontine snakes, species preying upon invertebrates are rare. Nevertheless, one speciose subclade of Central American Xenodontines feeds on earthworms and gastropods. França et al. [20] also studied the interaction between phylogeny and ecology in a tropical snake community, in open areas of the Brazilian Cerrado, concluding that the most important factor determining its structure was phylogeny. However, the authors suggested a strong ecological component that characterized a peculiar snake fauna.

All of these studies were conducted in speciose snake communities of tropical regions, such as the Brazilian Cerrado, Caatinga or humid Neotropical rainforests. However, a key question is whether this pattern is repeated in South American temperate snake communities, which are composed of fewer species in each of the representative clades. Despite the lower richness, temperate communities have representatives of Elapids (Elapidae), Viperids (Viperidae), and the three groups of colubrids that Cadle and Greene [1] proposed as forming the present-day Neotropical communities: Colubrines (Colubridae), Central American Xenodontinae, and South American Xenodontinae (both Xenodontinae groups comprise the Dipsadidae Neotropical endemic family proposed by Zaher et al. [21]). Therefore, these communities have an interesting and complex phylogenetic community structure that has not yet been thoroughly investigated. In this study, for the first time, we compare phylogenetic and ecological influences on diet (one of the main niche axis) in a temperate South American snake community.

## Materials and Methods

### Study Area

**Fig 1:**
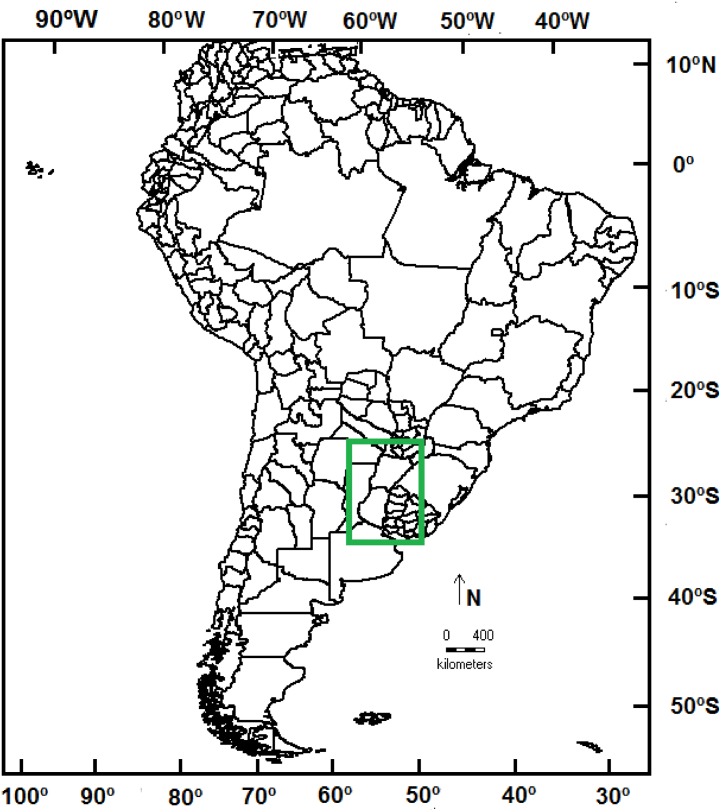
Study Area. Map of South America showing the temperate region under study.

The field study was carried out in an 800,000-ha study area in eastern Argentina, between 24° 41´ S to 35° 30´ S latitude and 62° 10´ W to 53° 15´W longitude (Fig 1). This region belongs to the Chacoan dominion (*sensu* Morrone [22]), and is characterized by a mosaic of vegetation ranging from wet savannas and grasslands to subtropical-temperate deciduous forests and a wide variety of wetlands. The geomorphology and landscape of this region have been strongly influenced by the three large South American rivers of the Plata Basin—the Paraná, Uruguay, and Paraguay Rivers—that converge to form the La Plata River. Cabrera [23] and Morrone [22] described phytogeographical and zoogeographical aspects of the region. The climate is seasonal, with a hot and rainy spring and summer (mean temperature: 25°C) and a cold and dry autumn and winter (mean temperature: 10°C, absolute minimum between -1°C and -6°C). Precipitation decreases from northeast to southeast, and annual precipitation ranges from 1000–1500 mm [24, 25].

### Ecological data collection

Dietary data were obtained by analyzing 1966 specimens pertaining to 25 species of snakes which belonged to 15 genera and represent four taxonomic families: Viperidae, Elapidae, Colubridae and Dipsadidae. We sampled the study area from January 1991 to April 2014, mainly by means of road sampling and time constrained searches (for details see Giraudo [26], López and Giraudo [27, 28], López et al. [29], Bellini et al. [8, 30], Giraudo et al. [31, 32]). The material was supplemented with original data from specimens deposited in the following scientific collections: Instituto Nacional de Limnología (INALI, Santa Fe), Museo Argentino de Ciencias Naturales “Bernardino Rivadavia” (MACN, Buenos Aires), Colección del Museo de La Plata (MLP, Buenos Aires), Museo Antonio Serrano (MAS, Entre Ríos), Universidad Nacional del Nordeste (UNNE, Corrientes), Museo provincial de Ciencias Naturales "Florentino Ameghino" (MFA, Santa Fe). We dissected the digestive tracts of each specimen to analyze their contents and identify prey items. The snout-vent lengths (SVL) of adult snakes were also measured. Snake diet was categorized into three groups according to the proportion of different prey items found, following Giraudo et al. [33]: specialist: over 70% of the diet is made up of the same item; trend towards specialization: an item represents between 50 and 70% of the diet; and generalist: no item accounts for more than 49% of the diet. For dietary analyses, we recognized eight discrete prey categories following previously published analyses about phylogenetic influence in snake communities [1, 7, 20]: hard-bodied invertebrates, soft-bodied invertebrates, fish, anurans, reptiles, birds, and mammals.

### Ethics Statement

We only collected recently road-killed snakes that were in good enough condition to extract data. No living specimens were collected or sacrificed. The field studies did not involve endangered or protected species. In Argentina, approval from the animal ethics committee is not required for research that does not involve experiments. All data collection adhered to the legal requirements of Argentina. The Secretaría de Medio Ambiente de la Provincia de Santa Fe, Secretaría de Ambiente de la Provincia de Entre Ríos, Subsecretaría de de Recursos Naturales y Medio Ambiente de la Provincia de Chaco, and the Ministerio de Ecología y Recursos Naturales Renovables de la Provincia de Misiones provided scientific collecting permits.

### Analysis

**Fig 2:**
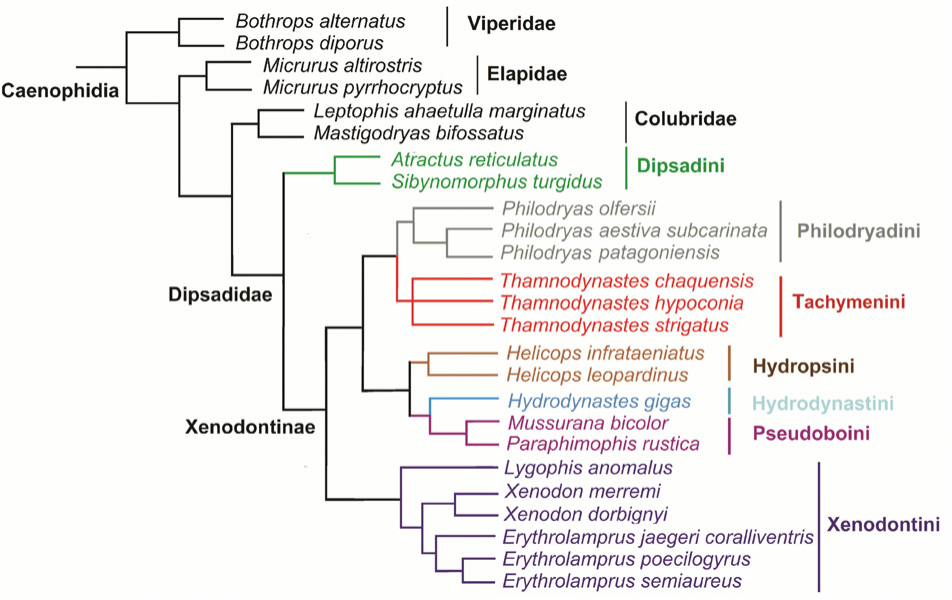
Phylogenetic relationships among 25 species of snakes used in the analysis of canonical phylogenetic ordination. Phylogeny based on Grazziotin et al. [36].

To detect a phylogenetic influence on diet, two ordination techniques were used: first, an indirect analysis and then a direct analysis. These two approaches are complementary and should both be used for a better understanding of the structure of the assemblage [34]. To assess the ecological influence we performed a principal component analysis (PCA), an indirect analysis, with the diet variables previously standardized. PCA was carried out with Infostat software version 5.1 [35]. To evaluate the role of history in structuring the assemblage, a direct analysis, a Canonical Phylogenetic Ordination (CPO), was used [36]. CPO is a modification of Canonical Correspondence Analysis (CCA) [37, 38,39], a constrained ordination method that promotes the ordination of a set of variables in such a way that association with a second set of variables is maximized. In our CPO, one of the matrices (Y) contained ecological data (diet) measured across all snake species in the assemblage, whereas the second matrix (X) consisted of a tree matrix that contained all clades in the assemblage, each coded separately as a binary variable. In the analysis where covariate were used, this matrix (E) contained ecological data (SVL, habitat use) that covary with the (Y) matrix. The CPO analysis thus consisted of finding the subset of groups (columns of X) that best explained the variation in Y, coupled with Monte Carlo permutations. Prior to the CPO analysis, we carried out the global permutation test to judge the significance of the relation between the diet and phylogeny. We performed CPO in CANOCO 4.5 for Windows, using CCA (a unimodal method that is the proper technique when data consist of frequencies with many zeros), symmetric scaling, biplot scaling, manual selection of environmental variables (monophyletic groups), 9,999 permutations, and unrestricted permutations. The manual selection consisted in testing each clade one by one to obtain *F*— and *P*—values. After each clade was tested, the significant clades were included in the model, the subsequent clade that most reduced the variance being tested and included if statistically significant (*P*<0.05). Since snake body size can affect the size and even type of prey ingested [32], we used the average SVL of adult males and females for each snake species as a covariate to minimize the effect of body size (S1 Table). We also used habitat use as covariate to test if this ecological attribute could influence the diet of our community of snakes. The diet data for the matrix (Y) were coded as frequencies of consumed prey in each category (S2 Table). The matrix of habitat use (E) was made with published information from field studies in the study area since 1991 [8, 26, 30–32, 40] (S1 Table). We recognized five discrete categories, defined by Cadle and Greene [1] and modified by Giraudo et al. [31], as follows: aquatic: dorsal and terminal displacement of eyes and nostrils, valvular closure of nasal cavity and/or mouth; arboreal: Small body mass (high length/mass ratio), compressed body, relatively long tail (sometimes prehensile), relatively large eyes, frequently enlarged vertebral scale row; fossorial: Small body size (length), reduced head width, scale reductions, small eyes, skull reinforcement, narrow snout, short tail; semi-aquatic: species mainly recorded in aquatic habitats and prey on aquatic animals but do not have the morphological modification of the aquatic species; terrestrial: generalized morphology. During our field observations, we recorded sympatry among all analyzed species. The phylogenetic matrix (X) combined phylogenies proposed by Castoe et al. [41], Fenwick et al. [42], Zaher et al. [21], Carrasco et al. [43] and Grazziotin et al. [44]. An illustration of the phylogenetic relationship of the studies species is shown in Fig 2. For *Bothrops* we considered a tribe what Carrasco et al. [43] considered a group.

## Results

**Fig 3:**
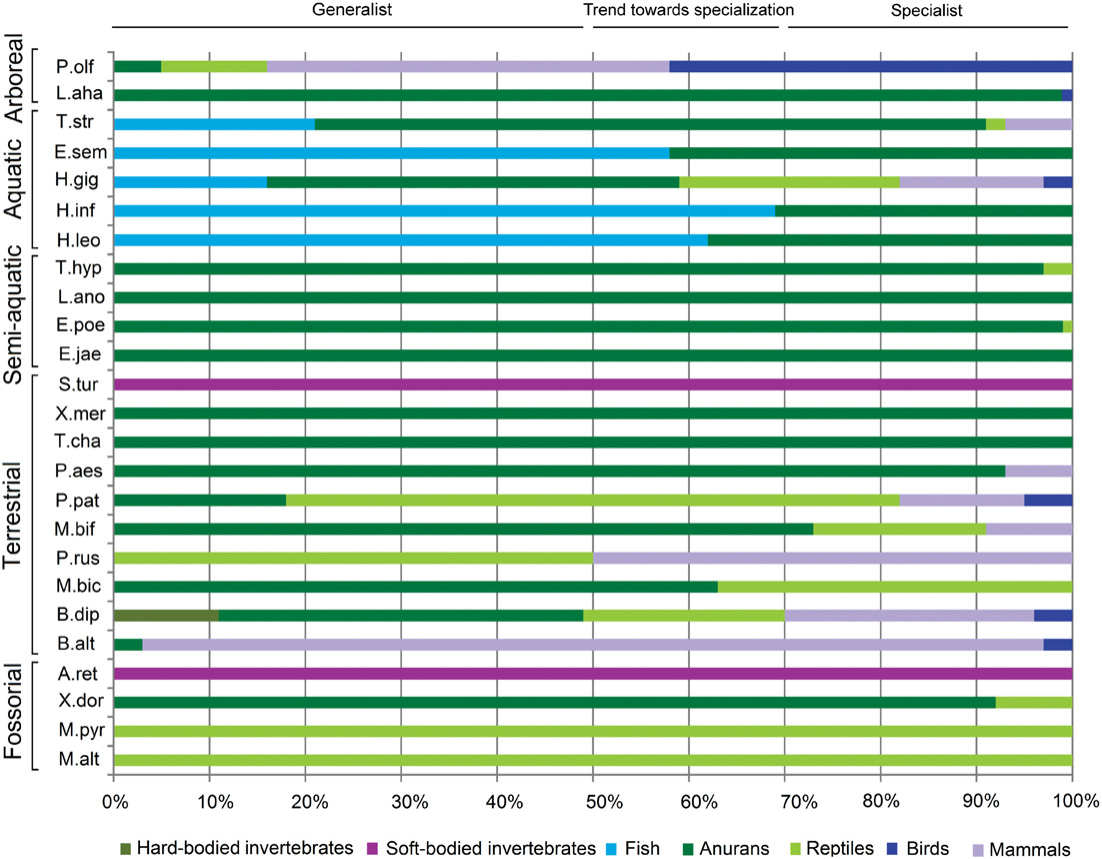
Diet of a community snake species from temperate South America, grouped according their habitat use. *Atractus reticulatus* (A. ret), *Bothrops alternatus* (B. alt), *Bothrops diporus* (B. dip), *Erythrolamprus jaegerii* (E. jae), *Erythrolamprus poecilogyrus* (E. poe), *Erythrolamprus semiaureus* (E. sem), *Helicops infrataeniatus* (H. inf), *Helicops leopardinus* (H. leo), *Hydrodynastes gigas* (H. gig), *Leptophis ahaetulla* (L. aha), *Lygophis anomalus* (L. ano), *Mastigodryas bifossatus* (M. bif), *Micrurus altirostris* (M. alt), *Micrurus pyrrhocryptus* (M. pyr), *Mussurana bicolor* (M. bic), *Paraphimophis rustica* (P. rus), *Philodryas patagoniensis* (P. pat), *Philodryas olfersii* (P. olf), *Philodryas aestiva* (P. aes), *Sibynomorphus turgidus* (S. tur), *Thamnodynastes chaquensis* (T. cha), *Thamnodynastes hypoconia* (T. hyp), *Thamnodynastes strigatus* (T. str), *Xenodon dorbingyi* (X. dor), *Xenodon merremii* (X. mer).

Snakes of our temperate community ate different kinds of prey that vary from invertebrates to higher vertebrates. Terrestrial species fed mainly on mammals, anurans, and reptiles, although in different proportions. Among them, *Bothrops diporus* and *Philodryas patagoniensis* were the species with more varied diets, the former eating a higher proportion of anurans and the latter more reptiles. *Mastigodryas bifossatus* and *Mussurana bicolor* consumed primarily anurans but also reptiles; the diet of *Paraphimophis rustica* consisted of reptiles and mammals in the same proportion. Five terrestrial species were specialist: *Ph*. *aestiva*, *Thamnodynastes chaquensis* and *Xenodon merremii* fed mostly on anurans, *B*. *alternatus* ate almost exclusively mammals, and *Sibynomorphus turgidus* only fed on soft-bodied invertebrates. Aquatic species (*Erythrolamprus semiaureus*, *Helicops infrataeniatus*, *H*. *leopardinus*) fed mainly on fish, and one species ate mostly anurans (*T*. *strigatus*). *Hydrodynastes gigas*, another aquatic snake, was the only generalist species in the assemblage, probably associated to its large body size, which enables it to consume a wide type of prey. In turn, most prey identified for aquatic snakes were exclusively aquatic or semi-aquatic species, which correlated to the type of habitat use (for details see: López and Giraudo [20]). On the other hand, semi-aquatic species (*E*. *jaegerii*, *E*. *poecilogyrus*, *L*. *anomalus* and *T*. *hypoconia*) fed almost exclusively on anurans. Among the arboreal species, *L*. *ahaetulla* consumed almost entirely arboreal anurans (*Hypsiboas* and *Scinax*) and *Ph*. *olfersii* preyed mostly on birds and mammals. Finally, of the fossorial species *M*. *altirostris* and *M*. *pyrrhocryptus* fed on elongated reptiles, *X*. *dorbingyi* fed on terrestrial anurans, and *A*. *reticulatus* ate soft-bodied invertebrates (Fig 3). Two axes extracted by the PCA explained 46% of diet variation in the snake assemblage. The first axis (28% of the variation) contrasted species that consumed anuran prey with those that preyed on mammals, reptiles and birds. The second axis (18% of the variation) contrasted species feeding on soft-bodied invertebrates with those that prey on anurans, mammals, birds and hard-bodied invertebrates.

The global permutation test, using CCA, demonstrated that the relation between the diet of the species and their evolutionary history (phylogeny) was highly significant (*F* = 3.796; *P* = 0.0010). In addition, the first canonical axis was not statistically significant (*F* = 4.506; *P* = 0.1615). Monte Carlo permutations from the canonical ordination revealed a significant phylogenetic effect on diet of species that make up the temperate snake communities studied. The analysis without using a covariate showed that phylogeny explained 81% of the variation in the diet, whereas 19% of the variation remained unexplained. The first two axes of this direct analysis explained 65.3% of the variation. The clade with the greatest variability was the subfamily Dipsadinae (39%), composed of two species, *Atractus reticulatus* and *Sybinomorphus turgidus*, which were the only species that fed on soft-bodied invertebrates (Table 1). Furthermore, this is the only subfamily in our temperate community that belongs to the Central American Xenodontines. The following four significant clades were genera. The first genus was *Micrurus* (15%), the second was *Helicops* (10%), then follows *Bothrops* (10%). The congeneric species generally had similar diets between them. The fourth genus, *Philodryas* (6%), comprises three species with different diets among them (Table 1). The last two clades contributing to significant dietary divergence were the tribes Alternatus (6%), which only included *B*. *alternatus*, and Pseudoboini (5%), to which *Mussurana bicolor* and *Paraphimophis rustica* belong (Table 1).

**Table 1:** Results of a phylogenetic ordination analysis using canonical correspondence analysis for the diets of 25 species in a snake community in temperate South America.

Taxa	Variation	Variation %	*F*	*P*
Dipsadinae	1	39	11.51	0.0035
*Micrurus*	0.4	15	5.59	0.0062
*Helicops*	0.27	10	5.47	0.0101
*Bothrops*	0.27	10	5.34	0.0122
*Philodryas*	0.15	6	3.25	0.0388
Alternatus	0.15	6	3.93	0.0394
Pseudoboini	0.13	5	4.01	0.0315

Clades are ranked by the amount of variation explained at each node. Percentage of the variation explained (relative to total unconstrained variation) and *F*- and *P*-values for each variable are given (9,999 permutations were used) for each main matrix. Note that no groups used for variable selection of variable yielded individual *P*≤0.05.

When adding habitat use as a covariate in the analysis, the diet variation explained by phylogeny dropped to 51%, whereas habitat use explained 17% of the variation and the remainder was unexplained. The first two axes of this analysis explained 83.1% of the total variation in diet. Again, Dipsadine (48%) was the clade that contributed most to diet variation (Table 2), with one fossorial (*A*. *reticulatus*) and othoneer terrestrial (*S*. *turgidus*) species. The other three clades that contributed significantly to explaining dietary variation were the tribe Alternatus (13%) and the genera *Micrurus* (10%) and *Leptophis* (7%) (Table 2). The first clade was composed of one terrestrial species; the second had two fossorial species, and the third included an arboreal species (*L*. *ahaetulla*) from the Colubridae.

**Table 2:** Results of a phylogenetic ordination analysis using canonical correspondence analysis for diets of 25 species in a snake community in temperate South America, using the use of habitat as a covariate.

Taxa	Variation	Variation %	*F*	*P*
Dipsadinae	0.93	48	16.68	0.0006
Alternatus	0.26	13	5.74	0.0305
*Micrurus*	0.19	10	5.11	0.0038
*Leptophis*	0.14	7	4.49	0.0196

Clades are ranked by the amount of variation explained at each node. Percentage of the variation explained (relative to total unconstrained variation) and *F*- and *P*-values for each variable are given (9,999 permutations were used) for each main matrix. Note that no groups used for variable selection yielded individual *P*≤0.05.

By contrast, the use of SVL as a covariate in the analysis resulted in phylogeny explaining 75% of the diet variation (almost as much as in the analysis with no covariate), with SVL explaining 17% of the variation and only 8% remaining unexplained (Table 3). The first two axes explained 63% of the variation. The clades that contributed significantly to the diversification of the diet were the same, and to the same extent, as in the analysis without covariate (Table 3). The subfamily Dipsadinae was always the clade that most contributed to dietary divergence, providing more than 35% of the variation in the three analyses.

**Table 3:** Results of a phylogenetic ordination analysis using canonical correspondence analysis for diets of 25 species in a snake community in temperate South America, using snake body size as a covariate.

Taxa	Variation	Variation %	*F*	*P*
Dipsadinae	0.89	36	10.44	0.0001
*Micrurus*	0.41	17	5.88	0.0063
*Helicops*	0.32	13	5.73	0.0063
*Bothrops*	0.24	10	5.11	0.0135
*Philodryas*	0.13	5	2.98	0.0526
Alternatus	0.14	6	3.95	0.0385
Pseudoboini	0.12	5	3.78	0.0373

Clades are ranked by the amount of variation explained at each node. Percentage of the variation explained (relative to total unconstrained variation) and *F*- and *P*-values for each variable are given (9,999 permutations were used) for each main matrix. Note that no groups used for variable selection yielded individual *P*≤0.05.

**Fig 4:**
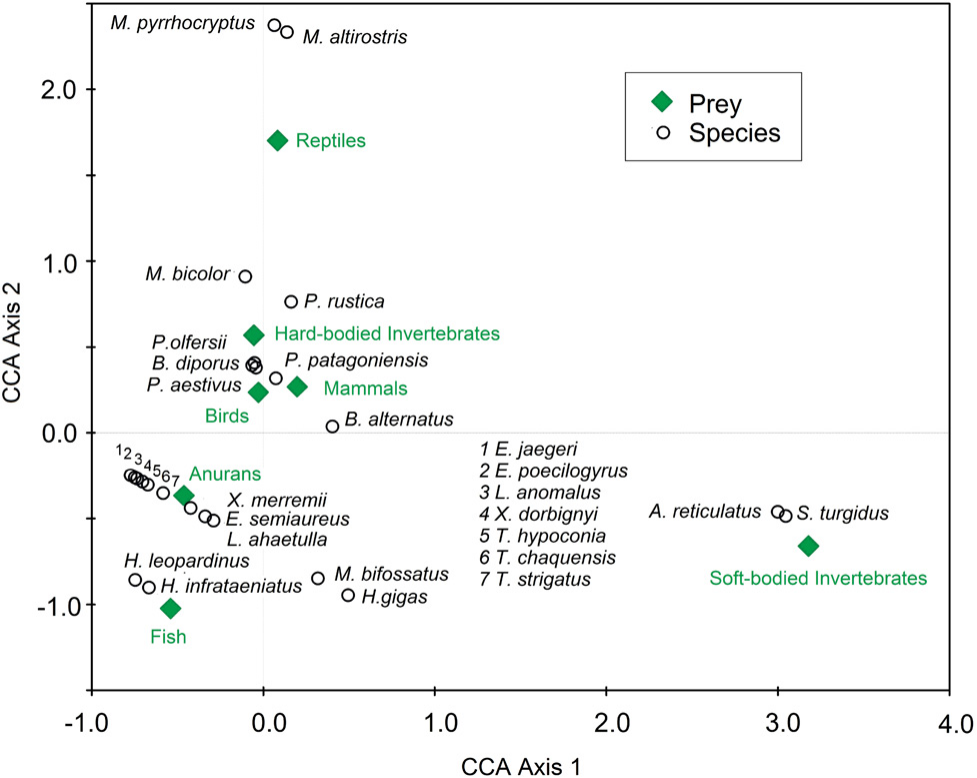
Biplot of snake diet from a phylogenetic ordination using canonical correspondence analysis (CCA). Canonical axes represent linear combinations of diets to snake phylogeny. Snake diet items are weighted averages of each species’ score. The first canonical axis accounts for 40% and the second canonical axis accounts for 23% of the total variation. Diet items (rhombus) close to the graph centre (0,0) indicate either low association with any snake clade (circles) or a positive association with a specific combination of all snake clades. Diet items displayed in the periphery of the graph indicate either high association with a specific snake clade or an occasional association, particularly for that clade with low occurrence (Ter Braak and Smilauer, 2002).

A plot of snake species scores on the two canonical axes describing dietary variation revealed how different the Dipsadinae subfamily was, because *Atractus reticulatus* and *Sybinomorphus turgidus* ate soft-bodied invertebrates, annelids (earthworms) and gastropod mollusk (snails and slugs), respectively (Fig 4). Another distinctive clade was the Elapids (composed of the *Micrurus* species) that appear at the top of the plot, whose species are specialists in eating reptiles. The remaining prey items group the other 21 species. The Viperids and two species of the tribe Philodryadini have independently converged to feed on terrestrial vertebrate prey, including mammals and reptiles, but in different proportions. On the other hand, most species of the Xenodontinae subfamily (South American Xenodontinae) fed on aquatic vertebrate prey, including frogs and fishes, though the phylogenetic origin of this type of diet was evident (Fig 3).

## Discussion

For Neotropical snakes, the ecological characteristics of individual assemblages are largely a function of the proportional representation of each lineage. Then the imprint of historical differences among the lineages emerges as a primary force molding the composition and ecological characteristics of any given assemblage [1]. The snake community of temperate South America studied here was composed of five lineages: Elapids, Viperids, Colubrines, Central American Xenodontinae (Dipsadinae) and South American Xenodontinae (Xenodontinae). Because every lineage that makes up a community carries some evolutionary baggage that permits only certain options in terms of resource use and ecological interactions, the present ecological relationships among species can only be understood in terms of historical patterns [1]. Furthermore, the structure of a community reflects the ecological interactions of the individuals who compose it, as well as their historical lineages [7, 20, 45]. In our study, this means that the diet of a species strongly depends on its evolutionary history, which reflects the clade to which it belongs. Our results suggest that the assemblage in the temperate snake community of South America we studied is, to a significant extent, the result of an admixture of evolutionarily independent lineages, each contributing a set of species with different diets. In other words, close relatives share more similarities in morphology, ecology, life history, and ecological niches than more distant relatives [46]. This tendency of species to retain ancestral ecological characteristic was called “phylogenetic niche conservatism” (PNC), and implies that related species differ less ecologically than might be expected if ecological diversification had occurred in an unconstrained manner [46–48]. Moreover, PNC can be defined as the trend of species to hold characteristics of their fundamental niche over time [46]. The ecological niche of diet seemed to be phylogenetically conservative in our community, because the species of each clade ate, at a general level, the same item preys: Viperids ate mostly mammals and some ectothermic prey, the Elapids had a diet based on elongated reptiles, and all three clades of Neotropical colubrids included species that consumed frogs and lizards. However, the various diets within each clade of the Xenodontines resulted in a low niche overlap, allowing the coexistence of different species of these two clades [1]. The similar diets among the species from the same subfamily, together with the results of phylogenetic analysis, are indicative of PNC of this ecological characteristic at the subfamily level. A study of three sympatric species of *Thamnodynastes* in the subtropical-temperate region of South America confirmed that their diets showed a high niche overlap [30]. Pianka [49] stated that sympatric species with high overlap along one dimension often show little overlap along other dimensions, and that resources can be shared if they are not in short supply. Anurans are indeed abundant in the area we studied. Xenodontine snakes preyed upon lizards, anurans and fish, but the South American clade of this group also preyed on mammals, birds and rarely ate invertebrates (which are consumed by some species of the Central American clade. The species of our assemblage belonging to the Central American Xenodontines (Dipsadinae), for example, fed only on soft-bodied invertebrates like earthworms and gastropods. This type of specialized diet was reported in other studies [50, 51] and, according to our results, this characteristic showed PNC. Additionally, in all CPO this subfamily was the clade that explained most of the variability in the community diet, which strongly validated the results. Recently diverged taxa tend to be ecologically similar, which shows a direct link between the evolutionary relatedness of organisms in a community, the characters they possess, and the ecological processes that determine their distribution and abundance [52]. In our temperate community, phylogenetically related snakes had more similar diets among them, as compared to species of different lineages that share their habitats. The particular diet of each clade seems to be independent of major habitat types. Moreover, the different diets are not likely to be the result of long-term evolutionary interactions between the lineages as each has evolved in separate biogeographical centers [1]. *H*. *gigas*, the two species of *Helicops* and the three *Thamnodynastes* species, for example, are aquatic, but they did not have the same type of diet. The first was a generalist eater, *Thamnodynastes* species were batrachophagous, and the *Helicops* species were mainly piscivorous. This reflects that the phylogeny was the most important factor influencing reptile assemblage; however, ecological factors may also be important, as other studies proved [1, 10, 20, 53]. Our results showed that diet is influenced more by phylogenetic history, although some ecological (habitat use) or morphological characteristics (SVL) can play a role. In semi-aquatic reptiles, there are generalist species, such as *H*. *gigas*, although aquatic or semi-aquatic prey are an important component of their diet [27, 32]. Some snakes that share the same habitat showed some diet convergences. The two arboreal snake species in our study, *Leptophis ahaetulla* and *Philodryas olfersii*, include prey such as arboreal amphibians (Hylidae), birds, and bird eggs in their diets [54]. Snakes that show morphological adaptations to live in the water, like *H*. *leopardinus*, *H*. *infrataeniatus* and *E*. *semiaureus*, mainly feed on fish (close to 60% of their diet), which are abundant, at least seasonally, in our study area. Semi-aquatic snakes of the genera *Erythrolamprus*, *Lygophis* and *Thamnodynastes* mainly feed on amphibians, and occasionally on fish. Around 80% of the analyzed species (20 out of 25 species) include amphibians in their diet. Ecological factors, such as the high abundance of amphibians in temperate South American wetlands, or phylogenetic factors, such as the preponderance of frog-eating species of the subfamily Xenodontine in the South American snake fauna, have been mentioned to explain this pattern [6, 31]. While most *Erythrolamprus* and *Lygophis* (previously *Liophis*) species are largely batrachophagous [31], phylogeny remained the major determinant in structuring this community of snakes. As to the ecological influence of the body size (SVL), we can highlight, for example, that the largest snake in our assemblage (*H*. gigas) was the one that had the most varied diet. Obviously, its larger body size allows it to include different types of prey in its diet, from fish to mammals [32]. When we used SVL as a covariate of snake diet, we found that phylogeny explained almost as much as in the analysis without the covariate, and that the unexplained variance was smaller than in any of the other tests. This may be because SVL seems to be a morphological feature that phylogenetically conserved, as shown by our results and those of others [1, 20, 31].

The effect of history is evident in our analysis, supporting the DHH. We found significant dietary differences among the clades that compose our assemblage due to phylogenetic causes. This pattern is particularly clear when comparing the variability explained by the first two axes in the indirect (PCA) and direct analyses (CPO). In the PCA, in which only the ecological influence of diet was assessed, these axes explained less than half of the variability. However, in the three analyses of CPO, phylogeny explained 60–80% of diet variability. This outcome supports the DHH, although it does not rule out the potential role of the CPH in the structure of the community, as suggested by the analysis with ecological characteristics (habitat use). As mentioned, these hypotheses are not mutually exclusive, and can act together to determine the structure of a community [7, 20]. We found that ecological factors accounted for some diet variation, but, in agreement with most studies conducted on reptile assemblages, the history of species best explained the variation [1, 7, 20, 45, 53]. The deep history of snakes appears to have played a profound role in determining their diet, and events that occurred in the remote past may have strongly influenced much of squamate biodiversity observed today [10]. Several studies have shown that snakes generally exhibit precise, genetically-determined, species-specific preferences for some prey types [14, 16, 17]. De Queiroz and Rodriguez-Robles [18] related the importance of snake chemosensory systems to diet specialization in their study of the origin of the egg eating in snakes. Their results suggest broad effects of predispositions on snake diets and thus illustrate how historical contingencies can shape the ecology of organisms [18]. This idea is what has led ecologists to consider the evolutionary history of organisms to help determine the underlying causes of the community structures we observe today [45].

We can now answer, at least in part, the question that Cadle and Greene [1] posed on what determines the organization of a community in time and space, pointing out that the community we studied has a deep phylogenetic effect that explains most of the variation in diet. Our analyses showed that the diet of the temperate snake community of South America is associated with past events in the evolutionary history of the group that molded the current feeding habits of the species in our assemblage.

## Supporting Information

S1 Table Average snout-vent lengths (SVL) of adult males and females and habitat use for each snake species used as a covariate in the analysis (matrix E). (DOCX)

S2 Table Diet data for the matrix (Y), coded as consumed prey category frequencies. Hard-bodied (H), Soft (S).
(DOCX)

## References

[1] Cadle JE, Greene HW. Phylogenetic patterns, biogeography, and the ecological structure of neotropical snake assemblages In: Ricklefs RE, Schluter D, editors. Species Diversity in Ecological Communities: Historical and Geographical Perspectives. Chicago: University of Chicago Press; 1993 pp. 281–293.

[2] Schoener TW. Resource partitioning in ecological communities. Science 1974; 185: 27–39. 17779277 10.1126/science.185.4145.27

[3] Winemiller KO, Pianka ER. Organization in natural assemblages of desert lizards and tropical fishes. Ecol Monogr 1990; 60: 27–55.

[4] Webb CO, Ackerly DD, Mcpeek MA, Donoghue MJ. Phylogenies and community ecology. Annu Review Ecol Evol Syst 2002; 33: 475–505.

[5] Cadle JE. The Neotropical colubrid snake fauna (Serpentes: Colubridae): lineage components and biogeography. Syst Zool 1985; 34: 1–20.

[6] Vitt LJ, Vangilder LD. Ecology of a snake community in northeastern Brazil. Amphibia—Reptilia 1983; 4: 273–296.

[7] Colston TJ, Costa GC, Vitt LJ. Snake diets and the deep history hypothesis. Biol J Linn Soc 2010; 101: 476–486.

[8] Bellini GP, Arzamendia V, Giraudo AR. Ecology of the viviparous snake *Thamnodynastes hypoconia* (Dipsadidae: Tachymenini) in Subtropical-temperate South America. Herpetologica 2013; 69: 67–79.

[9] Henderson RW, Dixon JR, Soini P. Resource partitioning in Amazonian snake communities. Contrib Biol Geol 1979; 22: 1–11

[10] Vitt LJ, Pianka ER. Deep history impacts present-day ecology and biodiversity. Proc Natl Acad Sci USA 2005; 102: 7877–7881. 15867150 10.1073/pnas.0501104102 PMC1142370

[11] Kembel SW. Disentangling niche and neutral influences on community assemblage: assessing the performance of community phylogenetic structure tests. Ecol lett 2009; 12: 949–60. 10.1111/j.1461-0248.2009.01354.x 19702749

[12] Greene HW. Snakes: the evolution of mystery in nature. Berkeley: California University Press; 2001.

[13] Luiselli L. Resource partitioning and interspecific competition in snakes: the search for general geographical and guild patterns. Oikos 2006; 114: 193–211.

[14] Burghardt GM. Comparative prey-attack studies in newborn snakes of the genus *Thamnophis* . Behaviour 1969; 33:77–114.

[15] Saviola AJ, McKenzie VJ, Chiszar D. Chemosensory responses to chemical and visual stimuli in five species of colubrid snakes. Acta Herpetologica 2012; 7: 91–103

[16] Burghardt GM, Layne DG, Konigsberg L. The genetics of dietary experience in a restricted natural population. Psych Science 2000; 11: 69–72. 10.1111/1467-9280.00217 11228846

[17] Ford NB, Burghardt GM. Perceptual mechanisms and the behavioral ecology of snakes In: Seigel RA, Collins JT, editors. Snakes: Ecology and evolutionary biology. New York: McGraw-Hill; 1993 pp. 117–164

[18] de Queiroz A, Rodríguez—Robles JA. Historical contingency and animal diets: the origins of egg eating in snakes. Am Nat 2006; 167: 684–694 16671012 10.1086/503118

[19] Emerson BC, Gillespie RG. Phylogenetic analysis of community assemblage and structure over space and time. Trends Ecol Evol 2008; 23: 619–30. 10.1016/j.tree.2008.07.005 18823678

[20] França FGR, Mesquita DO, Nogueira CC, Araújo AFB. Phylogeny and ecology determine morphological structure in a snake assemblage in the Central Brazilian Cerrado. Copeia 2008; 1: 23–38.

[21] Zaher H, Grazziotin FG, Cadle JE, Murphy RTW, Moura-Leite JC, Bonato SL. Molecular phylogeny of advanced snakes (Serpentes, Caenophidia) with an emphasis on South American Xenodontines: A revised classification and descriptions of new taxa. Pap Avuls Zool 2009; 49: 115–153.

[22] Morrone JJ. Cladistic biogeography of the Neotropical region: indentifying the main events in the diversification of the terrestrial biota. Cladistics 2014; 30: 202–214. 10.1111/cla.12039 34784690

[23] Cabrera AL. Regiones fitogeográficas argentinas. Encicl Arg Agric Jard 1994; 2: 1–85.

[24] Iglesias de Cuello A. Atlas físico de la República Argentina Buenos Aires: Atlas total de la República Argentina, Centro Editor de América Latina; 1982.

[25] Paoli C, Iriondo M, García N. Características de las cuencas de aporte In: Paoli Schreider M, editors. El río Paraná en su tramo Medio. Contribución al conocimiento y prácticas ingenieriles en un gran Río de Llanura. Santa Fe: Centro de Publicaciones, Secretaría de Extensión, Universidad Nacional del Litoral; 2000 pp. 27–68.

[26] Giraudo AR. Diversidad de Serpientes de la Selva Paranaense y del Chaco Húmedo Taxonomía, Biogeografía y Conservación. Buenos Aires: Literature of Latin America; 2001.

[27] López MS, Giraudo AR. Diet of the large water snake *Hydrodynastes gigas* (Colubridae) from northeast Argentina. Amphibia—Reptilia 2004; 25: 178–184.

[28] López MS, Giraudo AR. Ecology of the snake *Philodryas patagoniensis* (Serpentes, Colubridae) from Northeast Argentina. J Herpetol 2008; 42: 474–480. 10.1016/j.neuroimage.2008.05.040 18617423

[29] López MS, Giraudo AR, Arzamendia V, Chiaraviglio M. Biología reproductiva de la serpiente semiacuática *Liophis semiaureus* (Serpentes, Colubridae) en el nordeste de Argentina. Rev Chil Hist Nat 2009; 82:233–244.

[30] Bellini GP, Giraudo AR, Arzamendia V. Comparative ecology of three species of *Thamnodynastes* (Serpentes, Dipsadidae) in subtropical-temperate South America. Herpetol J 2014; 24: 87–96.

[31] Giraudo AR, Arzamendia V, López MS. Reptiles In: Parma J, Paggi JC, Iriondo M, editors. The Middle Paraná River: Limnology of a subtropical wetland. Berlin: Springer—Verlag; 2007 pp. 341–362.

[32] Giraudo AR, Arzamendia V, Bellini GP, Bessa CA, Costanzo MB. Ecology of the large South American snake, *Hydrodynastes gigas* (Serpentes: Dipsadidae). Rev Mex Biodivers 2014; 85: 1206–1216.

[33] Giraudo AR, Arzamendia V, Bellini GP, Bessa CA, Calamante CC, Cardozo G, et al Categorización del estado de conservación de las Serpientes de la República Argentina. Cuadernos de herpetología 2012; 26: 303–326.

[34] Lepš J, šmilauer P. Multivariate Analysis of Ecological Data České Budéjovice: Faculty of Biological Sciences, University of South Bohemia; 1999.

[35] Di Rienzo JA, Robledo CW, Balzarini MG, Casanoves F, Gonzalez L, Tablada M. InfoStat Software Estadístico. Córdoba: Universidad Nacional de Córdoba; 2005.

[36] Giannini NP. Canonical Phylogenetic Ordination. Syst Biol 2003; 52: 684–695. 14530135 10.1080/10635150390238888

[37] Ter Braak CJF. Canonical Correspondence Analysis: A New Eigenvector Technique for Multivariate Direct Gradient Analysis. Ecology 1986; 67:1167–1179

[38] Ter Braak CJF, Šmilauer P. CANOCO Reference Manual and User's Guide to Canoco for Windows: Software for Canonical Community Ordination (Version 4). Wageningen: Centre for Biometry; 1998.

[39] Shine R, Thomas J. Do lizards and snakes really differ in their ability to take large prey? A study of relative prey mass and feeding tactics in lizards. Oecologia 2005; 144: 492–498. 15891833 10.1007/s00442-005-0074-8

[40] Arzamendia V, Giraudo AR. Influence of large South American rivers of the Plata Basin on distributional patterns of tropical snakes: A panbiogeographical analysis. J Biogeogr 2009; 36: 1739–1749.

[41] Castoe TA, Smith EN, Brown RM, Parkinson CL. Higher-level phylogeny of Asian and American coral snakes, their placement within the Elapidae (Squamata), and the systematic affinities of the enigmatic Asian coral snake *Hemibungarus calligaster* (Wiegmann, 1834). Zool J Linn Soc 2007; 151: 809–831.

[42] Fenwick AM, Gutberlet RL Jr, Evans JA, Parkinson CL. Morphological and molecular evidence for phylogeny and classification of South American pitvipers, genera *Bothrops*, *Bothriopsis*, and *Bothrocophias* (Serpentes: Viperidae). Zool J Linn Soc 2009; 156: 617–640.

[43] Carrasco PA, Mattoni CI, Leynaud GC, Scrocchi GJ. Morphology, phylogeny and taxonomy of South American bothropoid pitvipers (Serpentes, Viperidae). Zool Scr 2012; 41: 109–124.

[44] Grazziotin FG, Zaher H, Murphy RW, Scrocchi GJ, Benavides MA, Zhang Y, et al Molecular phylogeny of the New World Dipsadidae (Serpentes: Colubroidea): A reappraisal. Cladistics 2012; 1: 1–23. 10.1111/j.1096-0031.2012.00393.x 34836446

[45] Vitt LJ, Pianka ER, Cooper WE, Schwenk K. History and the global ecology of squamate reptiles. Am Nat 2003; 162: 44–60. 12856236 10.1086/375172

[46] Cooper N, Jetz W, Freckleton RP. Phylogenetic comparative approaches for studying niche conservatism. J Evol Biol 2010; 23: 2529–2539. 10.1111/j.1420-9101.2010.02144.x 20964782

[47] Losos JB. Phylogenetic niche conservatism, phylogenetic signal and the relationship between phylogenetic relatedness and ecological similarity among species. Ecol Lett 2008; 11: 995–1003. 10.1111/j.1461-0248.2008.01229.x 18673385

[48] Wiens JJ, Ackerly DD, Allen AP, Anacker BL, Buckley LB, et al Niche conservatism as an emerging principle in ecology and conservation biology. Ecol Lett 2010; 13: 1310–1324. 10.1111/j.1461-0248.2010.01515.x 20649638

[49] Pianka ER. Niche overlap and diffuse competition. Proc Natl Acad Sci USA 1974; 71: 2141–2145. 4525324 10.1073/pnas.71.5.2141 PMC388403

[50] Carreira S. Alimentación de los Ofidios del Uruguay. Asociación Herpetológica Española, Monografías de Herpetología 2002; 6:1–126. 8403038

[51] Balestrin RL, Di-Bernardo M, Moreno AG. Feeding ecology of the neotropical worm snake *Atractus reticulatus* in southern Brazil. Herpetol J 2007; 17: 62–64.

[52] Kraft NJB, Cornwell WK, Webb CO, Ackerly DD. Trait evolution, community assemblage, and the phylogenetic structure of ecological communities. Am Nat 2007; 170: 271–283. 17874377 10.1086/519400

[53] Vitt LJ, Zani PA, Espósito MC. Historical ecology of Amazonian lizards: implications for community ecology. Oikos 1999; 286–294.

[54] López MS, Giraudo AR, Arzamendia V. *Leptophis ahaetulla marginatus*. Diet. Natural History. Herpetol Rev 2003; 34: 68–69.

