# Supplementary material for: Temperate Snake Community in South America: Is Diet Determined by Phylogeny or Ecology?
Source: PLoS One. 2015 May 6;10(5):e0123237. doi: 10.1371/journal.pone.0123237 (PMC4422434; doi:10.1371/journal.pone.0123237)
Supplement: S1 Table — (DOCX) [file pone.0123237.s001.docx]

**Table S1.** The average snout-vent lengths (SVL) of adult males and females and the habitat use for each snake species, use as a covariate in the analysis (matrix E).

|  | SVL | Habitat use |  |
| --- | --- | --- | --- |
| *Atractus reticulatus* | 267 | Fossorial |  |
| *Bothrops alternatus* | 835 | Terrestrial |  |
| *Bothrops diporus* | 678 | Terrestrial |  |
| *Erythrolamprus jaegerii* | 342 | Semi-aquatic |  |
| *Erythrolamprus poecilogyrus* | 361 | Semi-aquatic |  |
| *Erythrolamprus semiaureus* | 698 | Aquatic |  |
| *Helicops leopardinus* | 408 | Aquatic |  |
| *Helicops infrataeniatus* | 474 | Aquatic |  |
| *Hydrodynastes gigas* | 1379 | Aquatic |  |
| *Leptophis ahaetulla* | 737 | Arboreal |  |
| *Lygophis anomalus* | 373 | Semi-aquatic |  |
| *Mastigodryas biffosatus* | 1235 | Terrestrial |  |
| *Micrurus altirostris* | 653 | Fossorial |  |
| *Micrurus pyrrhocryptus* | 593 | Fossorial |  |
| *Mussurana bicolor* | 593 | Terrestrial |  |
| *Paraphimophis rustica* | 813 | Terrestrial |  |
| *Philodryas patagoniensis* | 796 | Terrestrial |  |
| *Philodryas olfersii* | 702 | Arboreal |  |
| *Philodryas aestiva* | 683 | Terrestrial |  |
| *Sibynomorphus turgidus* | 304 | Terrestrial |  |
| *Thamnodynastes chaquensis* | 424 | Terrestrial |  |
| *Thamnodynastes hypoconia* | 398 | Semi-aquatic |  |
| *Thamnodynastes strigatus* | 497 | Aquatic |  |
| *Xenodon dorbingyi* | 373 | Fossorial |  |
| *Xenodon merremii* | 628 | Terrestrial |  |
|  |  |  |  |
|  | | | |
