# Supplementary material for: Temperate Snake Community in South America: Is Diet Determined by Phylogeny or Ecology?
Source: PLoS One. 2015 May 6;10(5):e0123237. doi: 10.1371/journal.pone.0123237 (PMC4422434; doi:10.1371/journal.pone.0123237)
Supplement: S2 Table — Hard-bodied (H), Soft (S). (DOCX) [file pone.0123237.s002.docx]

**Table S2.** The diet data for the matrix (Y), coded as consumed prey categories frequencies.

| Species / Prey | H Invertebrates | S Invertebrates | Fishes | Amphibians | Reptiles | Birds | Mammals |
| --- | --- | --- | --- | --- | --- | --- | --- |
| *Atractus reticulatus* | 0 | 1 | 0 | 0 | 0 | 0 | 0 |
| *Bothrops alternatus* | 0 | 0 | 0 | 0.03 | 0 | 0.03 | 0.94 |
| *Bothrops diporus* | 0.11 | 0 | 0 | 0.38 | 0.21 | 0.04 | 0.26 |
| *Erythrolamprus jaegerii* | 0 | 0 | 0 | 1 | 0 | 0 | 0 |
| *Erythrolamprus poecilogyrus* | 0 | 0 | 0 | 0.99 | 0.01 | 0 | 0 |
| *Erythrolamprus semiaureus* | 0 | 0 | 0.58 | 0.42 | 0 | 0 | 0 |
| *Helicops leopardinus* | 0 | 0 | 0.62 | 0.38 | 0 | 0 | 0 |
| *Helicops infrataeniatus* | 0 | 0 | 0.69 | 0.31 | 0 | 0 | 0 |
| *Hydrodynastes gigas* | 0 | 0 | 0.16 | 0.43 | 0.23 | 0.03 | 0.15 |
| *Leptophis ahaetulla* | 0 | 0 | 0 | 0.9 | 0 | 0.1 | 0 |
| *Lygophis anomalus* | 0 | 0 | 0 | 1 | 0 | 0 | 0 |
| *Mastigodryas biffosatus* | 0 | 0 | 0 | 0.73 | 0.18 | 0 | 0.09 |
| *Micrurus altirostris* | 0 | 0 | 0 | 0 | 1 | 0 | 0 |
| *Micrurus pyrrhocryptus* | 0 | 0 | 0 | 0 | 1 | 0 | 0 |
| *Mussurana bicolor* | 0 | 0 | 0 | 0.63 | 0.37 | 0 | 0 |
| *Paraphimophis rustica* | 0 | 0 | 0 | 0 | 0.5 | 0 | 0.5 |
| *Philodryas patagoniensis* | 0 | 0 | 0 | 0.18 | 0.64 | 0.05 | 0.13 |
| *Philodryas olfersii* | 0 | 0 | 0 | 0.05 | 0.11 | 0.42 | 0.42 |
| *Philodryas aestiva* | 0 | 0 | 0 | 0.93 | 0 | 0 | 0.07 |
| *Sibynomorphus turgidus* | 0 | 1 | 0 | 0 | 0 | 0 | 0 |
| *Thamnodynastes chaquensis* | 0 | 0 | 0 | 1 | 0 | 0 | 0 |
| *Thamnodynastes hypoconia* | 0 | 0 | 0 | 0.97 | 0.03 | 0 | 0 |
| *Thamnodynastes strigatus* | 0 | 0 | 0.21 | 0.7 | 0.02 | 0 | 0.07 |
| *Xenodon dorbingyi* | 0 | 0 | 0 | 0.92 | 0.08 | 0 | 0 |
| *Xenodon merremii* | 0 | 0 | 0 | 1 | 0 | 0 | 0 |

References: Hard-bodied (H), Soft-bodied (S).
